# Clinical Cases

**Published:** 1893-02

**Authors:** Jennie Medley

**Affiliations:** Philadelphia, Pa.


### CLINICAL CASES.

JENNIE MEDLEY, M. D., PHILADELPHIA, PA.

CASE I.—Mrs. L., æt. sixty-two. This patient for two years past had been suffering with uterine hemorrhages. When she did not have a discharge of blood, had a constant discharge of greenish or yellowish offensive water, with an occasional cheesy lump. She had borne nine children, had ceased menstruating about her fifty-fifth year—but, within last two years flow has returned. For the last six months her health is failing; is naturally very active, but finds her strength rapidly giving way.

Symptoms.—Great heaviness in the lower extremities; tired all over; it is a great effort to go up-stairs; very low spirited; grieved because she could not do her housework; bowels constipated—must strain until weak before the bowels moved. Appetite poor; there were the burning, sticking pains through the cervix; on examination found left side of the cervix partially destroyed. Tried the speculum, but at all times the discharge of blood was so great that I could see nothing. Digital examination, however, revealed the condition. On the above symptoms she received a dose of Sil.$^{72m}$. In about a month she was in wonderfully better spirits, was very much stronger, and the offensive odor of the discharge had entirely disappeared. From a repetition of the 72m to the millionth within a year, of which she received two doses each, she suffered very little pain, the case apparently being at a standstill. Her principal suffering during this time was great mental depression. Until the end of six months after she received the last dose of Sil.$^{m}$ her symptoms remained the same; at the close of six months she begun to have dyspnœa; couldn't sleep in a room where the least heat had been admitted—must have windows open in addition to being in a cold room. She was still kept on S-l.; this state of affairs was permitted to go on for some days. Finally, was sent for in a great hurry; arrived at the house and learned that first one and then another member of the family had been taking their turn at fanning her all night. When they ceased to fan she gasped for breath; this with the fan-like motion of the alæ nasi led to a prescription of Carbo-veg.$^{cm}$ which in fifteen minutes relieved her; after six doses of Carbo-veg. at such intervals as were made necessary by the return of uncomfortable symptoms, the CM proved no longer palliative, a dose of the millionth of the same remedy was given. This relieved promptly. At the close of three weeks the symptoms had changed, the burning returned violently. Carbo-veg.$^{m}$ was again given without effect. In addition to the burning she had thirst, little and often; great restlessness; was thirsty, but a small quantity satisfied, and was followed by great distress in the stomach. Ars.$^{90m}$ was then given. I felt so sure that it would help that I left, thinking in a short time all would be well. The Ars. was administered at 1 P. M. Sunday, the next morning, at nine o'clock, I was sent for. She had passed the night in agony, and begged for Morphine. I went home, looked over the case, and sent her again a dose of the 72m of Silicea. It acted like magic. She fell asleep in one hour, and from that time on she had to be shaken up, to arouse her sufficiently to take nourishment. She had no pain and only complained of being so tired and sleepy. For twenty-four hours before she died she lay dead asleep, and died without pain. Her son, who is not a strict homœopath, could scarcely believe that one could die with such a disease with so little pain. The absence of odor also surprised him very much.

CASE II.—Mrs. W., æt. thirty-two, mother of four children, three months advanced in pregnancy. From the beginning of her pregnant condition had suffered with constipation, would be worn out after a stool, she was obliged to go to bed; during the three months of pregnancy didn't have what she would call a good movement. At last she took la grippe which Rhus-t., the 1m, helped in twenty-four hours. She felt so well that she was going to get up, when suddenly she was taken with a hemorrhage, not violent, but very steady; the blood was bright red with dark clots. She had no pain, but terrific nausea. She had been in this condition two hours when I arrived. I immediately gave her a dose of Ipecac., but it neither brought on pains nor stopped the hemorrhage, so all to be done was to wait. The hemorrhage continued and still symptoms did not change. After several hours of watching and waiting she became weak, a profuse cold sweat covered her body, wanted to make her will immediately, would not be persuaded not to. Acon.$^{2c}$ was given in a broken dose, three teaspoonfuls of water every fifteen minutes; pains come on and the fœtus and all, which was of five weeks' development at most, was expelled. She was now exceedingly weak and immediately after delivery I gave a dose of Sul.$^{cm}$. After several hours she became violently thirsty. She dropped to sleep and in a few minutes woke up with violent pain in the heart. This made me think of Lach., but felt I would be sure of it. I then waited until she fell asleep again. She awoke in the same manner. I then gave her Lach. This relieved her. After taking the Lach. she again slept, but it was only for a very short time, and the pain was still quite severe but less sharp. Two of my brother physicians suggested remedies: one suggested Sepia and the other Pyrogen. Having given Sepia myself in the beginning for the constipation, I knew it would be of no avail now, and I felt certain that Pyrogen would be the remedy to pull her through if it were possible. Accordingly, after watching the temperature gradually rise to 101½°, eight hours after the Lach. I gave Pyrogen$^{50m}$; that was about 2 P. M. At 5 P. M. she urinated naturally. One hour later she had a large stool, the first hard lump was removed with the fingers, she was too weak to strain it away. Two hours later she had another soft stool without the least trouble. When the examination was first made at the beginning of the abortion the rectum was piled full of hard stool. These two symptoms encouraged me very much; the Pyrogen wore out every seven hours, which was also the experience of the physician who suggested it. After several doses of the 50m had been given the cm was given the patient. In four days the patient was completely out of danger and in three weeks was sitting up. In exactly four weeks after the abortion menstruation came on with a hemorrhage at twelve o'clock at night. She lost about a quart and a half of blood; this was also full of dark clots. The same physician who suggested the Pyrogen now gave Ipecac. I called several hours after, but the hemorrhage still continued. I gave Pyrogen, which stopped the hemorrhage and the patient made a good recovery. After the first six hours during the abortion I censured myself for not using mechanical measures, but now I am satisfied, as I feel quite sure I would have had a post-partum hemorrhage that I could not manage, being ignorant of how closely Pyrogen resembled Ipecac. in that respect. I also feel satisfied that the patient would have died had she not received Pyrogen, as the only case of septicemia I have ever lost was one which presented just such symptoms.

---

The following committee was appointed upon the subject of Vaccination to report at the next meeting:

Drs. Close, Custis, and A. G. Allan.

